# Protocol for a parallel economic evaluation of a trial comparing two surgical strategies in severe complicated intra-abdominal sepsis: the COOL-cost study

**DOI:** 10.1186/s13017-020-00294-4

**Published:** 2020-02-21

**Authors:** Joshua S. Ng-Kamstra, Elissa Rennert-May, Jessica McKee, Skyla Lundgren, Braden Manns, Andrew W. Kirkpatrick

**Affiliations:** 1 Department of Critical Care Medicine, University of Calgary, 2500 University Drive NW, Calgary, Alberta T2N 1 N4 Canada; 2 Department of Medicine, Section of Infectious Diseases, University of Calgary, Calgary, Canada; 3 Department of Surgery, University of Calgary, Calgary, Canada; 4 Surgical Services, Alberta Health Services, Calgary, Canada; 5 Department of Medicine, University of Calgary, Calgary, Canada; 6 Department of Community Health Sciences, University of Calgary, Calgary, Canada; 7 The Trauma Program, University of Calgary, Calgary, Canada

**Keywords:** Cost-effectiveness analysis, Cost-utility analysis, Quality of life, Randomized controlled trial, Intra-abdominal infections, Sepsis, Laparotomy

## Abstract

**Background:**

The risk of death in severe complicated intra-abdominal sepsis (SCIAS) remains high despite decades of surgical and antimicrobial research. New management strategies are required to improve outcomes. The Closed Or Open after Laparotomy (COOL) trial investigates an open-abdomen (OA) approach with active negative pressure peritoneal therapy. This therapy is hypothesized to better manage peritoneal bacterial contamination, drain inflammatory ascites, and reduce the risk of intra-abdominal hypertension leading to improved survival and decreased complications. The total costs and cost-effectiveness of this therapy (as compared with standard fascial closure) are unknown.

**Methods:**

We propose a parallel cost-utility analysis of this intervention to be conducted alongside the 1-year trial, extrapolating beyond that using decision analysis. Using resource use metrics (e.g., length of stay, re-admissions) from patients at all study sites and microcosting data from patients enrolled in Calgary, Alberta, the mean cost difference between treatment arms will be established from a publicly-funded health care payer perspective. Quality of life will be measured at 6 months and 1 year postoperatively with the Euroqol EQ-5D-5 L and SF-36 surveys. A within-trial analysis will establish cost and utility at 1 year, using a bootstrapping approach to provide confidence intervals around an estimated incremental cost-effectiveness ratio. If neither operative strategy is economically dominant, Markov modeling will be used to extrapolate the cost per quality-adjusted life years gained to 2-, 5-, 10-year, and lifetime horizons. Future costs and benefits will be discounted at 1.5% per annum. A cost-effectiveness acceptability curve will be generated using Monte Carlo simulation. If all trial outcomes are similar, the primary analysis will default to a cost-minimization approach. Subgroup analysis will be carried out for patients with and without septic shock at presentation, and for patients whose initial APACHE II scores are > 20 versus ≤ 20.

**Discussion:**

In addition to an estimate of the clinical effectiveness of an OA approach for SCIAS, an understanding of its cost effectiveness will be required prior to its adoption in any resource-constrained environment. We will estimate this key parameter for use by clinicians and policymakers.

**Trial Registration:**

ClinicalTrials.gov, NCT03163095, registered May 22, 2017.

## Background

Severe complicated intra-abdominal sepsis (SCIAS) imparts a mortality risk of 30-40% when individuals present in shock, even with the most advanced care [1–3]. For those who survive, hospitalization is often prolonged and fraught with complications including respiratory failure, renal failure, major cardiac events, wound infections, deep space infections, thromboembolic disease, neurocognitive dysfunction, and prolonged weakness. The high incidence of SCIAS compounds the complexity of care required to treat the disease and its complications, creating a large resource burden for health systems globally. Estimates of the cost of standard care per case in the Netherlands was 86,077 USD in 2010; in Austria, the effective cost “per survivor” was reported as 232,400 USD in 1998 [4, 5].

After initial surgery for SCIAS, in the absence of an absolute indication to leave the abdomen open (for example, bowel left in discontinuity), the fascia is usually closed definitively. With this approach, repeat unplanned laparotomy is commonly required to establish surgical source control [6]. Early closure of the fascia can also lead to abdominal compartment syndrome with impairment of ventilation and renal perfusion.

An alternate strategy of leaving the fascia open (“open abdomen”, OA) with active negative pressure peritoneal therapy (ANPPT) is being studied in an international, multisite-randomized controlled trial (Closed Or Open after Laparotomy, COOL); the comparator is standard fascial closure at the initial operation [7]. Investigators hypothesize that ANPPT will allow for ongoing drainage of infected, inflammatory peritoneal fluid, decreasing the systemic propagation of inflammatory mediators [8, 9]. OA will also facilitate repeat operative washouts; these advantages may improve survival. Both approaches are acceptable options for the management of SCIAS according to World Society of Emergency Surgery guidelines [10, 11]. Despite varied expert opinion on the merits of the OA approach, an examination of the evidence base reveals persistent equipoise. Even if an OA strategy demonstrates clinical benefit in this trial, costs may be significantly higher in this approach due to increased need for critical care resources, including mechanical ventilation, while the fascia is open [4]. Alternatively, costs may be lower if OA with ANPPT results in rapid resolution of systemic inflammation and a shortened duration of critical illness.

If the OA strategy shows clinical benefit, the resources required to adopt it into practice must be accurately counted; each resource used, including operating room and ICU time, comes with an opportunity cost (i.e., less resources available for other medical treatments). We therefore propose a 1-year prospective cost-utility analysis with robust quality of life valuation to be performed alongside this RCT, using decision analysis to extrapolate beyond 1 year if required. Since the economic implications of this strategy may be large, determining the incremental cost effectiveness ratio of this alternate therapy is important to guide adoption in any resource-constrained health care environment.

## Objectives

### Primary Objective

The primary objective of this analysis is to estimate the incremental cost-effectiveness ratio of the open abdomen (OA) approach versus fascial closure for SCIAS over the 1-year time horizon of the COOL trial. Resource use data will be requested from all study sites, and total cost estimates will be established based on unit costs derived from microcosting data from Calgary, Alberta.

If all outcomes are similar in the COOL trial, the analysis will instead default to a cost-minimization approach.

### Secondary objectives

Secondarily, we aim to determine mean total cost difference for OA versus primary fascial closure for both the overall cohort and for pre-specified subgroups of patients including:

1. Patients with and without the presence of septic shock at the time of initial surgery
2. APACHE II score > 20 or ≤ 20

Further, we will assess quality of life (QOL) after surgical management of SCIAS, identifying determinants of poor quality of life across the study population and quantifying differences in QOL between the two treatment arms.

Finally, if neither operative strategy is dominant (i.e., if greater costs and improved outcomes accrue in one treatment arm), we will conduct a Markov analysis to determine the cost per quality-adjusted life years gained over a lifetime horizon. We will estimate the incremental cost-effectiveness ratio and create a cost-effectiveness acceptability curve using Monte Carlo simulation.

## Methods

### COOL study

The methodology of the COOL trial has been published elsewhere [7] and is briefly summarized here. To be included in the trial, adult patients will have complicated intra-abdominal infection (purulent, feculent, or enteric contents in the peritoneal cavity at the time of operation) and present with severe disease (either septic shock, World Society of Emergency Surgery Sepsis Severity Score ≥ 8, or a Calgary Predisposition-Infection-Response-Organ dysfunction score ≥ 3). Patients will be excluded if presenting during pregnancy, if there is a perceived inability to close the abdomen safely without inducing intra-abdominal hypertension, or if there is an absolute indication for “damage control laparotomy,” among other exclusion criteria. Patients in the *intervention* arm of the trial will have the abdomen temporarily closed with an ABTHERA^TM^ device with planned repeat operation 24-72 h later. In the *control* arm, the fascia will be closed in the usual fashion after a closed-suction intraperitoneal drain is placed. Randomization will be performed online after confirming eligibility, with a permuted block randomization strategy to ensure close balance between treatment arms at each site.

### Population for COOL-cost

For the primary cost analysis, the patient population will include all patients randomized to open abdomen (OA) or primary fascial closure in the COOL trial.

Data on resource use will be requested from all participating sites. Microcosting data from Calgary, Alberta, will be used to establish unit costs and develop estimated cost totals.

### Identification, measurement, and valuation of resource use

All costs that may differ between study arms will be considered from a publicly-funded health care payer perspective using a microcosting approach where possible (Table 1). Costs can be divided into those associated with the index hospitalization, follow-up care, any required readmission or delayed inpatient surgical procedure, and day medicine and surgery encounters (Table 2).

**Table 1:** Microcosting in the Calgary zone, Alberta Health Services

*Microcosting in Alberta*	
To determine how costs differ between treatment arms in this analysis, we will use microcosting [12] data from hospitals in the Calgary Zone of Alberta Health Services. Resources used in the management of each patient will be enumerated, valued, and summed to create a highly precise cost-of-care for each individual. Less intensive methodologies using average patient costs for similar groups would be insensitive to the differences in cost between the open abdomen and primary fascial closure arms.	
Microcosting is made possible by patient-specific resource use data collected by Alberta Health Services and calculated in accordance with national Management Information Systems guidelines [13]. First, the quantity of physical materials and diagnostics necessary for the care of each patient are tracked. These include medications, blood products, patient-traceable disposables, radiologic investigations, and laboratory tests. The quantity of each is multiplied by the unit cost (estimated annually) to provide a total cost estimate and these are summed. The direct human resources required for care including nursing and support staff are quantified and similarly costed. Physician billing fees are added to this to create a total cost of labor. The indirect costs of the spaces and systems of care (e.g., ICU bed or OR time) are then apportioned to each patient [12]. Costs across these domains are summed to create an estimate of cost for each patient encounter. Outpatient encounter data are also available to estimate the cost of follow-up with specialists, rehabilitation, and outpatient services such as dialysis.	
The availability and quality of cost data reporting in Alberta is highly regarded and has previously been used in the assessment of surgical costs [14]. Our primary analysis will combine resource use data from all COOL sites with microcosting data from Calgary to create estimates of cost in each treatment arm.

**Table 2:** Microcosting data items required from Calgary, Alberta sites

Variable	Description	Data required	Data source
*Baseline/Admission data*			
Patient unique identifier	Single datum permitting linkage across multiple databases	Unique Lifetime Identifier	COOL trial
Hospital at enrolment	Hospital where initial laparotomy is performed	Site name	COOL trial
Patient postal code	Approximate location of patient’s domicile (necessary for calculating transportation costs)	Postal code	COOL trial
Patient age	In years	Age in years	COOL trial
Patient sex	Male, female, other	Sex	COOL trial
ICD-10 admission diagnosis	Single admission diagnosis	ICD-10 code	COOL trial
Shock at the time of initial OR	Hypotension requiring pressors for MAP > 65 or serum lactate > 2 mmol/L after resuscitation	Yes/No	COOL trial
Admission APACHE II score	Severity-of-disease classification score	Score at time of admission	COOL trial
*Index hospitalization*			
Operating room			
Dates of each operating room encounter	During index hospitalization	List of dates	COOL trial
Procedures performed at each operating room encounter	All procedure codes included	List of procedure codes	COOL trial
Duration of all operating room encounters	Documented entry into and exit from operating room	Time in minutes	COOL trial
Anesthetist billings associated with each operating room encounter	Total physician claims	Billings in CAD	Alberta Medical Association (AMA) fee schedule
Surgeon billings associated with each operating room encounter	Total physician claims	Billings in CAD	AMA fee schedule
Operating room drug costs	List, quantity, and cost of all medications used during surgery	Medications and costs	Finance oracle microcosting database (Oracle)
List and quantity of disposables used at each surgical encounter	All non-sterilizable surgical tools designed for one-time use, as well as suture materials and dressings	List and quantity	Oracle
Surgical instrument sets used at each surgical encounter	Name of each set used (e.g., major abdominal)	List of instrument sets	Oracle
Postoperative care			
Length of stay in ICU and cost	Stay in a designated critical care environment	Length in days	COOL trial, Oracle
Length of stay on general ward and cost	Length of hospital stay, less the length of ICU stay	Length in days	COOL trial, Oracle
Length of stay in post-anesthesia care unit	Stay in a designated PACU	Length in days	Oracle
Direct and indirect costs of PACU stay	Itemized cost of PACU stay including personnel, disposables, medications, and overhead costs	Costs in CAD	Oracle
Physician billings associated with hospital stay	Total Alberta Health billings, less surgeon, and anesthetist operative billings	Billings in CAD	AHS data analytics (DIMR)
Postoperative diagnostic imaging costs	Total of per-study fees, plus the AHS billings of diagnostic radiologists	Costs in CAD	Oracle
Postoperative percutaneous procedure costs	Total of per-procedure fees, plus the AHS billings of interventional radiologists	Costs in CAD	Oracle
Costs of laboratory testing	Total in-hospital laboratory testing, less total study-specific laboratory testing costs	Costs in CAD	Oracle
Inpatient drug costs	Total pharmaceutical costs	Costs in CAD	Oracle
Cost of blood products used	Total cost of in-hospital blood product use	Costs in CAD	Oracle
Cost of nursing and allied health care not included in per-diem unit cost	Total cost of specialized RN and other allied health providers	Costs in CAD	Oracle
Cost of disposables used in postoperative patient care	Total cost of disposables	Costs in CAD	Oracle
*Follow-up care*			
Number of surgeon visits (to 1 year)	Regardless of reason for visit	Number of visits	DIMR
Number of other specialist physician visits (to 1 year)	Regardless of reason for visit	Number of visits	DIMR
Total outpatient AHS billings	Regardless of reason for services	Billings in CAD	DIMR
*Readmissions*			
Number and length of each readmission within 1 year	Regardless of reason for readmission	Number and length in days	DIMR
Total hospital cost, including physician billings, associated with each readmission	Regardless of reason for visit	Costs in CAD	DIMR
*Subsequent OR costs*			
Dates of each operating room encounter within 1 year	After index hospitalization	List of dates	COOL trial
Procedures performed at each operating room encounter	All procedure codes included	List of procedure codes	COOL trial
Duration of all operating room encounters	Documented entry into and exit from operating room	Time in minutes	COOL trial
Anesthetist billings associated with each operating room encounter	Total physician claims	Billings in CAD	AMA Fee Schedule
Surgeon billings associated with each operating room encounter	Total physician claims	Billings in CAD	AMA Fee Schedule
List and quantity of disposables used at each surgical encounter	All non-sterilizable surgical tools designed for one-time use, as well as suture materials and dressings	List and quantity	Oracle
Surgical instrument sets used at each surgical encounter	Name of each set used (e.g., major abdominal)	List of instrument sets	Oracle
*Ambulatory care costs*			
Number, type, and cost of all other ambulatory encounters (other than family physician and specialist encounters)	All day medicine and day surgery encounters (including dialysis) within one year	Number, type, and costs in CAD	DIMR
*Costs to patients and caregivers*			
Cost of transportation to and from family physician and specialist appointments	Total estimated costs	Number of visits, approximate distance to domicile	DIMR
*Productivity costs*			
Number of days absent from work at 1 year	Self-reported number of days absent from work	Total days	COOL trial
*Outcomes*			
Mortality at 90 days	Primary outcome	Dichotomous variable	COOL trial
Mortality at 1 year	Long-term survival	Dichotomous variable	COOL trial
Presence of a stoma at initial hospital discharge	Ileostomy or colostomy	Dichotomous variable	COOL trial
Presence of a stoma at 90 days and 1 year	Ileostomy or colostomy	Dichotomous variable	COOL trial
EQ-5D-5 L and SF-36 at 90 days	Validated quality of life measure	Values at 90 days	COOL trial
EQ-5D-5 L and SF-36 at 1 year	Validated quality of life measure	Values at one year	COOL trial

A secondary analysis from a societal perspective will be conducted if the data permit. This will include nonmedical and patient-borne costs attributable to the illness and associated care, and the value of lost productivity.

#### Index hospitalization costs

First, we will consider the costs of surgery for the alternate strategies. The number of minutes spent in the operating room, care by surgeons and anesthesiologists, and the use of sterilizable surgical tools will be valued and included. The cost of surgical disposables will be included; while temporary abdominal closure devices may be provided free-of-charge by the manufacturer for use in the trial, the market value of these devices will be determined and included.

Postoperatively, the cost of care provided in the post-anesthesia care unit, intensive care unit (ICU), and the general ward will be determined and included. The costs of care in ICU are hypothesized to represent a large proportion of the inpatient costs for SCIAS patients and may drive differences in cost between the two treatment arms. In the Canadian context, ICU care costs are approximately three times higher than that on a general ward, and so ICU length of stay and costs will be specifically examined in this analysis [15].

The microcosting approach will provide data on the cost of nursing care, diagnostic imaging, percutaneous interventions, laboratory testing (excluding additional testing performed solely for trial purposes), medications including antibiotics, blood products, additional care provided by other health providers including physiotherapy, occupational therapy, and enterostomal therapists, and the costs of disposables required for care in hospital. Furthermore, data will be provided on indirect costs such as patient transport, housekeeping, administration, and building maintenance. These costs will be summed and included in the primary analysis.

#### Follow-up care costs

We will include the costs of follow-up with specialist physicians and enterostomal therapists, the management of wound infections, time spent in a rehabilitation facility, and the cost of any ongoing organ support such as hemodialysis for renal failure.

#### Readmissions

If readmission to hospital within one year is required for any reason, this will be recorded. Given that the relevance of a readmission to the original illness is difficult to determine, the full cost of readmissions will be included in the primary analysis. In a secondary analysis, if it is possible to determine which admissions (or portions thereof) are unrelated to the original illness, these will be excluded.

#### Surgical procedures after initial discharge

All surgical procedures within 1 year will be costed and included in the primary analysis. In secondary analysis, only surgeries related to the diagnosis of SCIAS will be included, which might include reversal of an enterostomy, management of an enterocutaneous fistula, or management of an abdominal hernia.

#### Ambulatory case costing

We will identify, cost, and include all related outpatient day surgery, day medicine, and emergency room visits occurring after discharge.

#### Costs to patients and caregivers

After discharge from hospital, the cost of transportation to and from health care providers will be estimated for each patient by multiplying the number of follow-up visits by the distance traveled to and from the listed home address and using a standard per-kilometer cost value. These costs will be included in secondary analysis from a societal perspective.

#### Productivity costs

Absence from paid work after a diagnosis of SCIAS may have significant economic consequences. The number of days absent from paid work after discharge will be tabulated for all individuals less than 65 years of age, and the value of this absence will be calculated using a friction cost methodology and included in secondary analysis [16].

### Quality of life

Quality of life data are being collected in the COOL trial as a secondary outcome, using the Euroqol EQ-5D-5 L and SF-36 surveys at 6 months and 1 year postoperatively. Utility values will be estimated using the EQ-5D-5 L index score, using the visual analog score as a secondary analysis. We will assess quality of life data across the study population to identify drivers of good or poor quality of life at the 6-month and 1-year mark. We will then quantify differences in quality of life between the OA and fascial closure arms.

### Cost-effectiveness analysis alongside the trial in year 1

Total quality-adjusted life years at the 1-year mark will be determined for each individual in the trial using mortality and QoL data. The incremental cost-effectiveness ratio (ICER) will be calculated as:

$$ \mathrm{ICER}=\frac{C_{\mathrm{OA}}-{C}_{\mathrm{PFC}}\ }{Q_{\mathrm{OA}}-{Q}_{\mathrm{PFC}}} $$

where *C*_OA_ is the mean cost of the open abdomen strategy, *C*_PFC_ is the mean cost in the standard-of-care primary fascial closure strategy, *Q*_OA_ is the value of quality-adjusted life years (QALYs) associated with surgery with an open abdomen strategy, and *Q*_PFC_ is the value of QALYs associated with the primary fascial closure strategy.

The ICER will be expressed in 2020 CAD per QALY. A bootstrapping approach using sampling with replacement will be used to create an overall estimate of the ICER with confidence intervals based on 1000 samples from the study population.

### Modeling of cost-effectiveness beyond 1 year

If neither the OA or fascial closure strategy is economically dominant (i.e., better outcomes but also higher costs accruing in one treatment arm), Markov modeling will be used to estimate cost, QALYs, and cost per QALY gained at the 2-, 5-, 10-year, and lifetime horizons.

To perform this analysis, a set of mutually exclusive, collectively exhaustive health states after surgery for SCIAS will be determined. These might include, for example, complete recovery, recovery with ileostomy or colostomy, chronic dependence on renal replacement therapy, neurologic impairment due to stroke or complications of critical illness, and death. The transition probabilities between these health states will be estimated using existing literature, COOL study data, and locally available datasets. A cycle length of 1 year will be used. Each health state will be assigned utility values. Across a simulated population of patients, total QALYs gained and additional costs accrued (from, for example, further surgery), as well as the cost per QALY, will be estimated. Future costs and benefits will be discounted at 1.5% per annum.

Monte Carlo simulation will then be used to determine a cost-effectiveness acceptability curve.

### Sample size and power

A limitation in conducting a cost-effectiveness analysis in the context of an RCT is that trials are powered to demonstrate differences in clinical outcome and not necessarily differences in cost between treatment arms. In this case, power will also be limited by costing resources within the population of patients recruited in Calgary, Canada—meaning less variability in costing estimates.

This limitation in power will be mitigated by using extensive sensitivity and scenario analyses including the above bootstrapping approach to define a cost effectiveness acceptability curve.

### Compliance with reporting guidelines and methodological literature

This analysis follows CADTH (Canadian Agency for Drugs and Technologies in Health) guidelines for the economic evaluation of health technologies [17]. The reference case we plan to use is a cost-utility analysis. The intervention and its standard-of-care comparator are clearly delineated, and the setting for the economic analysis has been established. We will use a publicly-funded health care payer perspective over a lifetime horizon, with future discounting of costs and benefits at 1.5%. Subgroups with potentially differing costs and benefits have been prespecified. We will include all relevant costs; effectiveness, including quality of life valuation, will be provided by the outcomes of the COOL trial. The results of this analysis will be reported following existing guidelines.

## Discussion

The COOL trial is being conducted in centers around the world, giving rise to a diverse study population and clinically generalizable results. We plan to incorporate all available data on health resource use from global sites, and, combined with unit cost data from the Calgary microcosting environment, establish estimates of total cost for each treatment arm. This approach accounts for differing health resource use at all study sites while creating a single estimate of the ICER that can be used by clinicians and hospital leaders in evaluating the OA strategy. However, it does not account for how the economic context might differ between the countries and centers in which the trial is being conducted; unit costs of specific health resources may differ significantly. For resources that are found to be the main drivers of cost, we will therefore obtain unit costs from all sites and conduct extensive sensitivity analyses. Through these sensitivity analyses, we will create a more complete picture of the incremental cost-effectiveness ratio in individual sites where costs and even outcomes may differ.

## Conclusions

To date, the cost of managing patients using an open-abdomen ANPPT approach compared with managing patients using SCIAS remains unknown. The COOL trial has begun to recruit participants and full accrual is anticipated by December, 2023. If the trial demonstrates improved outcomes with an OA strategy, accurate estimation of the cost-effectiveness of this approach will be necessary prior to its widespread adoption. Our proposed analysis will address this critical question.

## Data Availability

Not applicable

## Abbreviations

ANPPT: Active negative pressure peritoneal therapy
CADTH: Canadian Agency for Drugs and Technologies in Health
COOL: Closed Or Open after Laparotomy
ICER: Incremental cost-effectiveness ratio
OA: Open abdomen
QALY: Quality-adjusted life year
QOL: Quality of life
SCIAS: Severe complicated intra-abdominal sepsis
